# The Timeline of the Association Between Diabetes and Cardiovascular Diseases: A Narrative Review

**DOI:** 10.3390/jcm14196877

**Published:** 2025-09-28

**Authors:** Silvia Ana Luca, Raluca Malina Bungau, Sandra Lazar, Andreea Herascu, Laura Gaita, Vlad-Florian Avram, Bogdan Timar

**Affiliations:** 1 Doctoral School of Medicine, “Victor Babes” University of Medicine and Pharmacy, 300041 Timișoara, Romania; silvia.luca@umft.ro (S.A.L.); andreea.herascu@umft.ro (A.H.); 2 Department of Cardiology, “Victor Babes” University of Medicine and Pharmacy, 300041 Timisoara, Romania; 3 Centre for Molecular Research in Nephrology and Vascular Disease, “Victor Babes” University of Medicine and Pharmacy, 300041 Timisoara, Romania; gaita.laura@umft.ro (L.G.); avram.vlad@umft.ro (V.-F.A.); bogdan.timar@umft.ro (B.T.); 4 Department of Diabetes, “Pius Brinzeu” Emergency Hospital, 300736 Timisoara, Romania; malina.bungau@umft.ro; 5 First Department of Internal Medicine, “Victor Babes” University of Medicine and Pharmacy, 300041 Timisoara, Romania; 6 Second Department of Internal Medicine, “Victor Babes” University of Medicine and Pharmacy, 300041 Timisoara, Romania

**Keywords:** type 2 diabetes mellitus, cardiovascular diseases, metabolic syndrome, cardiovascular outcome trials, cardiovascular risk

## Abstract

Diabetes and cardiovascular diseases (CVDs) are two strongly associated conditions that mutually influence each other. This review aims to follow the historical timeline of their association by highlighting the changing paradigm in CV risk management and treatment strategies in type 2 diabetes (T2D). While the discovery of insulin was a breakthrough in reducing life-threatening complications like diabetic ketoacidosis, patients with diabetes still faced a poor prognosis in terms of macrovascular outcomes, especially CVDs. Initial efforts in improving outcomes by tightly controlling glycemia proved insufficient, highlighting the complex relationship between the two diseases. After decades of focusing solely on glucose-lowering strategies, rosiglitazone, a promising new drug was developed, ultimately raising the flag about the potential higher risk of CV complications like myocardial infarction associated with its use. This turning point shifted the focus towards CV safety of novel glucose-lowering drugs, mandating for the development of cardiovascular outcome trials. Several drug classes, like sodium-glucose co-transporter-2 inhibitors (SGLT2i) and glucagon-like peptide-1 receptor agonists (GLP-1 RAs), exceeded expectations by not only providing safety but also benefits in patients with T2D and CVDs and becoming the new standard of care in T2D management. The historical evidence linking T2D and CVDs has shaped regulatory requirements for cardiovascular outcome trials, guideline recommendations, and current therapeutic strategies. These insights highlight the importance of early interventions and a multidisciplinary approach to optimize patient outcomes.

## 1. Introduction

In the modern era, the link between cardiovascular disease (CVD) and diabetes, specifically T2D, is well-established and widely recognized, with similar risk factors like age, smoking, lifestyle, and obesity being incriminated in the pathophysiology of both diseases. Insulin resistance leads to impaired lipid metabolism and atherogenic dyslipidemia, while hyperglycemia is a driver for oxidative stress, inflammation, and endothelial dysfunction, collectively contributing to both the initiation and progression of CVDs [1]. In recent years, diabetes has become an emerging public health issue, its prevalence having one of the most important increases among chronic diseases in the past 40 years [2]. According to an estimation by the World Health Organization, the number of people with diabetes worldwide was 171 million in the year 2000 [3], increasing to 588.7 million in 2025 [4], and with numbers continuing to rise alarmingly.

CVDs are the leading cause of morbidity and mortality in this population, with recent epidemiological data indicating that patients with T2D have a 73% higher risk of myocardial infarction (MI) and an 84% higher risk of heart failure (HF) when compared to individuals without diabetes [4]; comprehensive studies show that the presence of diabetes approximately doubles the risk of CVDs, underscoring the need to elucidate the relationship between type 2 diabetes (T2D) and CVDs to guide targeted management and prevention [5,6].

By following the historical context and progress in researching the association between these two conditions, the primary objective of this narrative review is to summarize the historical timeline that links type 2 diabetes and CVDs by highlighting the paradigm shifts in treatment strategies and their implications in current practice. We performed a literature search in PubMed, Embase, Scopus, and Web of Science for publications in English from recent decades, landmark studies from earlier years, and the latest relevant research articles to provide a wider historical context. Articles were selected based on their relevance and were presented in a narrative form to highlight the evolving landscape in T2D and CVD management.

## 2. The Beginning: Diabetes and Cardiovascular Diseases

The first clues regarding possible associations between CVDs and diabetes, with an emphasis on T2D, were first mentioned in the early 20th century [7], with previous descriptions of diabetes including observations about potential complications in these patients, but without any documented specific links to CVDs [8].

The year 1921 marks an important step in the treatment of diabetes, with the discovery of insulin which dramatically reduced mortality rates and the risk of life-threatening complications like diabetic ketoacidosis [9]. However, as the lifespan of these patients substantially improved, chronic complications became more frequent, and despite managing glycemia, patients with diabetes were still at an increased risk of developing CVDs when compared to the nondiabetic population, indicating a more complex relationship between CV complications and diabetes that was not just a consequence of poor blood glucose control, as initially believed [10].

In one of its first clinical observations from the 1930s, Elliott P. Joslin, one of the pioneers of diabetes treatment, noted that patients with diabetes were more susceptible to arteriosclerosis and was thus among the first to suggest a possible connection between diabetes and vascular complications [11].

The Framingham Heart Study, initiated in 1948, was the first evidence-based, large-scale research that provided insights into the relationship between CVDs and diabetes [12]. As a landmark longitudinal study, it provided strong evidence that diabetes significantly increases the risk of developing CVDs, particularly coronary artery disease (CAD) and stroke, and that these patients had a 3-fold increase in CV mortality rates, therefore highlighting the importance of CV risk management in patients with diabetes and shifting the link between these two conditions from clinical observations to validated facts [13].

The UK Prospective Diabetes Study (UKPSD) was another large-scale trial conducted between 1977 and 1997 that demonstrated, like the Framingham study, how intensive blood glucose control reduced microvascular complication rates [14]. However, while results showed a potential benefit in terms of macrovascular outcomes like CV events, they lacked statistical significance, suggesting that additional factors besides glucose control should be considered in reducing the risk of CVDs in T2D patients [15].

Over the years, landmark studies have reinforced the importance of managing CV risk in patients with diabetes [16] by expanding the knowledge of how diabetes contributes to CVDs, along with emphasizing the role of early interventions in reducing microvascular outcomes, and shaping current treatment strategies towards a centered approach on CV risk reduction [17] and ultimately challenging the initial concept of “the lower, the better” in terms of glycemic control.

## 3. Metabolic Syndrome (MeS): An Evolving Definition

Obesity was historically seen as sign of good health, affluence, and fertility, reflecting major changes in diet and cultural perceptions [18]. Previously viewed as a marker of affluence, obesity has transformed in the modern era into the “globesity” epidemic, representing both a major public health challenge and a significant economic strain on nations across the globe, with its impact spanning all age ranges and socioeconomic groups [19].

The earliest historical representation of obesity is the famous Venus of Willendorf, a Paleolithic figurine dating from 24,000 BCE [20]. Centuries later, during the Baroque era, we encounter the same full-figure women, now known as the Rubensians, reflecting the historical standards, where fuller body types were associated with a higher socioeconomic status, beauty, and health [21]. It is noteworthy that a physique once idealized as the epitome of beauty and health is now regarded as a potential health hazard.

A link between obesity and metabolic disorders has always existed, yet scientific correlations between the two conditions have only been documented in the past few centuries. In the 18th century, Italian anatomist Giovanni Batista Morgagni, through his meticulous autopsies, observed and documented various pathological conditions associated with excess visceral fat [22].

The 20th century marks significant changes in understanding the link between obesity, metabolic disorders, and CV risk factors. In the 1920s, Austrian physicians Karl Hitzenberger and Martin Richter-Quittner made clinical observations that contributed to the understanding of the relationship between diabetes and hypertension: Hitzenberger observed that patients with diabetes often displayed associated high blood pressure while Richter’s research highlighted that hypertension in diabetic patients could lead to further complications like vascular damage and CVDs [23,24]. In 1923, Eskil Kylin describes a condition that linked high blood pressure, high blood sugar, obesity, and hyperuricemia, the “hypertoni-hyperglikemi-hyperurikemi syndrom”, contributing significantly to the early conceptualization of what now is known as metabolic syndrome [25]. Himsworth’s 1936 landmark paper “Diabetes Mellitus: Its Differentiation into Insulin-sensitive and Insulin-insensitive Types” was helpful in shaping the understanding of diabetes, with his research suggesting that insulin resistance was a key mechanism in the development of the disease, but also a significant risk factor for diabetes, particularly T2D [26]. In the 1940s, French physician Jean Vague introduced the concept of android obesity and proposed that the distribution of body fat was an important factor in the development of complications such as diabetes, atherosclerosis, gout, and kidney stones. His theory, first introduced in 1947, highlighted the impact of fat distribution in predicting metabolic and CV risk and was formally published almost a decade later [27]. In the mid-20th century, the concept of the “trisyndrome métabolique” proposed by Jean Camus described the presence of obesity, hyperlipidemia, and hyperglycemia and is considered one of the earliest attempts to link metabolic disorders with an increased risk of CVDs. The term “metabolic syndrome” (MeS) was first used in 1977 by Haller to describe a cluster of conditions like obesity, diabetes, hyperlipoproteinemia, hyperuricemia, and hepatic steatosis as contributing factors to the development and progression of atherosclerosis [28]. In 1988, Dr. Gerald Reaven introduced the concept of “Syndrome X” to describe a cluster of conditions like insulin resistance, dyslipidemia, and hypertension that increase CV risk [29]. One year later, Kaplan expanded on this concept by introducing the term “deadly quartet” and adding obesity to the list of CV risk factors [30].

In 1998, the World Health Organization (WHO) began to formalize the concept of MeS [31]. The first definition of MeS focused on the presence of insulin resistance along with one of the following criteria: hypertension, hypertriglyceridemia or hypo-HDL cholesterolemia, obesity, and microalbuminuria. Shortly after, the European Group for the Study of Insulin Resistance (EGIR) introduced its own set of criteria for MeS that was like the WHO definition but did not include microalbuminuria [32]. In 2001, the National Cholesterol Education Program Adult Treatment Panel III (ATP III) introduced the revised criteria for diagnosing MeS, which no longer required the presence of specific factors like insulin resistance, but instead, the diagnosis was based on the presence of three out of five of the following factors: abdominal obesity, hypertriglyceridemia, reduced HDL-cholesterol, elevated BP, and impaired fasting glucose levels or T2D [33]. Further refinements of the 2005 International Diabetes Federation (IDF) definition placed a greater emphasis on central obesity and using different waist circumference thresholds for men and women. In addition to central obesity, the IDF definition requires the additional presence of two of the following criteria: elevated triglyceride levels, low HDLc levels, high blood pressure, and fasting plasma glucose levels greater than 100 mg/dL [34]. In 2009, a joint consensus regarding the definition of MeS agreed on standard definition that requires the presence of at least three of the following criteria: waist circumference (ethnic-specific), hypertriglyceridemia, or lipid-lowering treatment, HDLc < 40 mg/dL for men and <50 mg/dL for women, or lipid-lowering treatment [35].

According to epidemiological data, the prevalence of MeS varies significantly worldwide depending on the diagnostic definition used: 12.5% with the ATP III criteria and 28.2% with the IDF definition [36]. However, the changing definitions of MeS differ in both diagnostic criteria and cut-off values, and faced criticism because of the inconsistencies in prevalence estimates, ultimately affecting patient classification and potentially influencing CV risk assessment and treatment decisions [37].

## 4. The Past: The Lower, the Better

As evidence of the strong link between hyperglycemia and CVDs emerged, it became essential to conduct large-scale studies to address key questions about the relationship between the management of diabetes and the occurrence of its complications [38]. Over the years, landmark diabetes trials have provided valuable insights into the long-term effects of glycemic control on the prevention of diabetes-related complications and further highlighting the importance of CV risk reduction in this high-risk population [39].

The United Kingdom Prospective Diabetes Study (UKPDS) evaluated the impact of conventional versus intensive glucose control on diabetes-related complications in 5120 patients with newly diagnosed T2D. Despite the obvious benefits in reducing microvascular complications, it failed to demonstrate a significant reduction in CAD or overall CV events during the initial follow-up period [14,40]. However, extended follow-up studies later showed that intensive glycemic control was associated with a considerable reduction in the risk of MI and all-cause mortality, postulating a so-called “legacy effect” in which early blood glucose control may have long-lasting benefits [15].

The Diabetes Control and Complications Trial (DCCT) examined the effect of near-normal blood glucose levels on the progression of microvascular events in patients with type 1 diabetes (T1D). While intensive therapy delayed the onset and slowed the progression of microvascular complications, establishing the importance of tight glycemic control in people with T1D, it also raised concerns about the potential impact of an increased risk of severe hypoglycemia. After the trial ended, the Epidemiology of Diabetes Interventions and Complications (EDIC) study was initiated to evaluate the long-term effects of DCCT’s intensive versus conventional glucose control on both micro- and macrovascular complications. One of the most significant findings from EDIC is the concept of “metabolic memory” or the “legacy effect”, showing that the benefits of intensive glucose control in reducing diabetes-related complications like nephropathy were maintained [41].

The Action to Control Cardiovascular Risk in Diabetes (ACCORD) study aimed to determine whether achieving normal HbA1c levels (<6%) through intensive therapy would lower CV events such as MI, stroke, or CV death in T2D patients who had established CVDs or additional CV risk factors versus standard therapy (HbA1c level between 7% and 7.9%). The study included several additional secondary analyses beyond the impact of glycemic control and CV outcomes, like lipid-lowering therapy, blood pressure control, progression of diabetic retinopathy, and effects of glucose control on cognitive impairment. In the glycemia subanalysis, results showed that intensive therapy not only failed to reduce CV events but also led to increased mortality rates in an already vulnerable population, ultimately leading to the early termination of the trial. These results pointed towards a potential harm associated with aggressive glucose control, underscoring that the “one fits all” approach is not the best strategy when it comes to high-risk patients. Another subanalysis compared the benefit of adding fibrate to standard statin therapy alone on reducing fatal and non-fatal CV events, but results were unsuccessful, without additional benefits seen in the fibrate group. The blood pressure subanalysis assessed whether targeting a systolic blood pressure of less that 120 mmHg would reduce CV events compared to standard blood pressure control (targeting <140 mmHg systolic), which did not result in a significant overall reduction in the primary composite cardiovascular endpoint [42]. The ACCORDION study, a follow-up to the ACCORD trial, confirmed the lack of long-term CV benefits following the intensive glucose control strategy [43].

Another hallmark trial, the Action in Diabetes and Vascular Disease: Preterax and Diamicron MR Controlled Evaluation (ADVANCE) trial, investigated whether intensive glucose control using gliclazide MR and other glucose-lowering medications, alongside intensive blood pressure control aiming for a target systolic blood pressure < 130 mmHg, would lead to improved cardiovascular outcomes in T2D patients compared to standard care. Results showed that intensive glucose control reduced the risk of microvascular complications but without a significant impact on the rate of major CV events, mainly due to limited statistical power and modest difference in HbA1c levels between the two study groups, while intensive blood pressure control led to a significant reduction in both microvascular and macrovascular outcomes [44].

The Veterans Affair Diabetes Trial (VADT) (2009) showed similar results regarding intensive glycemic control in participants with poorly controlled T2D, with no significant impact found on the rates of major CV events, mortality, or the progression of microvascular complications. Shockingly, a higher incidence of adverse events, particularly hypoglycemia, was observed in the intensive therapy arm. Along with the ACCORD and ADVANCE trials, VADT examined individuals with long-standing T2D and found similar results regarding intensive glucose control. All three trials demonstrated a lack of cardiovascular benefits from intensive glycemic control in these populations [45]. Intensive glucose control may increase the risk of severe hypoglycemic episodes, which can lead to adrenergic stimulation, endothelial dysfunction, and inflammation, or even promote arrhythmias in these patients, mechanisms that may help explain the lack of benefits on macrovascular outcomes observed in the landmark studies [46].

The outcomes of the hallmark diabetes trials raised an important question regarding the role of tight glycemic control in reducing macrovascular complications, demonstrating that the relationship between diabetes and CVD is complex and not solely attributed to hyperglycemia. This historical background brings valuable insights and reinforces the idea that a comprehensive approach is needed in managing CVD in this high-risk population [39].

## 5. The Great Paradigm Shift

For decades, the treatment of diabetes focused mainly on achieving good glycemic control. After the emergence of data coming primarily from the hallmark randomized controlled trials (RCTs), it became clear that managing diabetes should also heavily focus on reducing the risk of macrovascular complications, particularly CVDs [47]. Until 2007, CV safety was not the primary goal of studies on glucose-lowering drugs, since it was well-established that hyperglycemia was a major risk factor for diabetes-related complications [48]. Rosiglitazone, an antidiabetic drug that works primarily by increasing the sensitivity of target cells to insulin, decreasing HbA1c levels, with a relatively low risk of hypoglycemia and a significant improvement in insulin resistance, was initially approved for medical use in 1999. What seemed to be a potential groundbreaking drug raised safety concerns regarding fluid retention, especially when used in combination with insulin [49]. Shortly after approval, reports suggested that rosiglitazone, along with other drugs from the thiazolidinedione class, can induce HF, making the FDA issue warnings about this potential side effect and thus highlighting the need for additional long-term studies to address increasing concerns about the drug’s CV safety [50]. In 2006, A Diabetes Outcome Progression Trial (ADOPT) [51] was published, and although rosiglitazone improved glycemic control with the lowest incidence of monotherapy failure compared to glyburide and metformin, its use was associated with higher rates of bone fractures and fluid retention. While the study was not specifically designed to assess CV outcomes, it continued to drive concerns regarding the drug’s CV safety. The debate was even more amplified with the release of another large, randomized trial, DREAM, which showed a significantly higher risk of HF in patients treated with rosiglitazone [52].

A major change in the treatment of diabetes occurred in 2007, with the publication of Nissen’s meta-analysis which aimed to evaluate the risk of MI and CV death associated with the use of rosiglitazone by pooling data from 42 clinical trials. Results were alarming: rosiglitazone was associated with a 43% increased risk of MI and potentially CV deaths [53]. Later that year, the FDA issued a black-box warning regarding rosiglitazone’s risk of ischemic events, causing a dramatic decline in usage and prescription of the drug [54]. Following Nissen’s meta-analysis, GSK, the manufacturer of rosiglitazone, published the RECORD trial, a study designed to assess the CV safety of the drug. Results confirmed an increased risk of HF, but inconclusive data regarding the increased risk of MI [55]. However, the trial faced serious criticism for methodological issues, and the FDA decided to restrict the use of the drug, and while most of these restrictions were lifted in 2013 [56], rosiglitazone remains rarely used in clinical practice. Following the controversy, strict regulations regarding the occurrence of major adverse cardiovascular events (MACEs) in newly introduced diabetes medications were implemented, mandating pharmaceutical companies to conduct long-term cardiovascular outcome trials (CVOTs) that evaluate the CV safety of the drugs prior to their approval [57].

So, what was the role of these CVOTs and why were they so necessary? In terms of study design, their main endpoint was to prove the CV safety of glucose-lowering drugs, much-needed in a population already at an increased risk of experiencing negative outcomes. Prior to the 2008 FDA guidance [54], the drug approval process tested mainly HbA1c as the primary endpoint for efficacy and required a relatively small sample size, while CV adverse events that occurred during the trials were not independently adjudicated. As for patient selection, participants usually had an overall lower CV risk: younger and with a shorter disease duration, a study population that does not accurately reflect the reality. One of the most important requirements of CVOTs is the inclusion of high-risk participants: older, with more advanced disease and a higher burden of comorbidities or organ damage.

Additionally, CV safety was established at an upper bound of the 95% confidence interval (CI) for a risk ratio (RR) of major CV events of below 1.3. If the data from the pre-approval application falls below this threshold, a post-marketing CV is not required. However, if the upper bound of the two-sided 95% CI for the estimated RR falls between 1.3 and 1.8, and the overall risk-benefit evaluation favors approval, a post-marketing trial will be necessary. An RR greater than 1.8 indicates a significantly increased risk, i.e., inferiority of the drug and therefore no approval. New requirements also mandate that CV events (MI, stroke rates, and CV mortality, along with other potential endpoints) to be adjudicated by an independent committee in a blinded manner during all phase 2 and 3 clinical trials. CVOTs require a minimum duration of 2 years to provide data on the long-term CV safety of glucose-lowering drugs, requirements that lead to longer development programs and the inclusion of a larger cohort consisting mainly of high-risk patients [58,59].

## 6. The Present: Safety First

Driven by concerns about the thiazolidinedione class, specifically rosiglitazone, the FDA issued new regulations regarding the assessment of long-term CV safety of all new glucose-lowering medications [57]. Along with glycemic control and overall safety, newer T2DM medications need long-term CVOTs that demonstrate at least CV neutrality, if not CV benefits. Key requirements include an evaluation of a composite of major cardiovascular events (MACEs) as primary endpoint, along with the noninferiority of the drug compared to placebo [60]. This marked a paradigm shift in the development of diabetes treatment, with drugs no longer being approved solely on their glucose-lowering effect.

**Figure 1:**
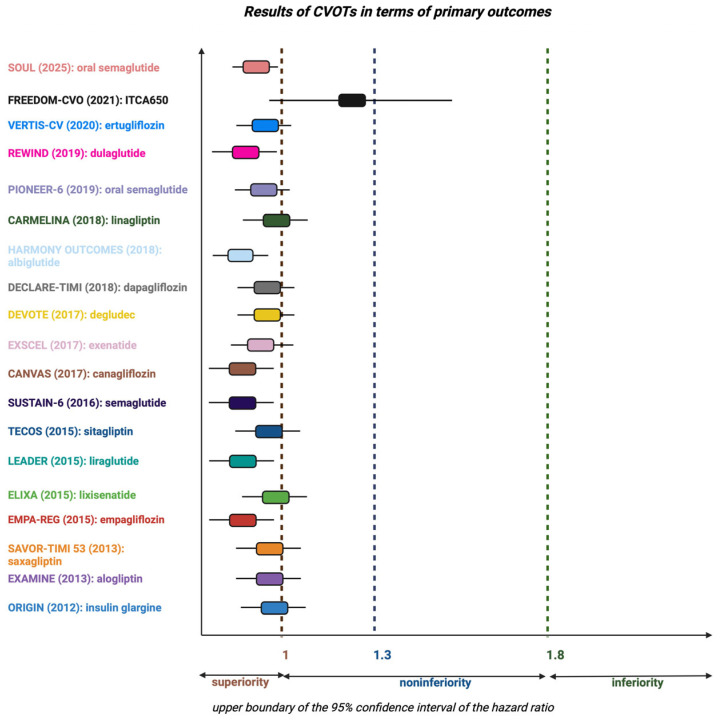
Results of CVOTs in terms of primary outcomes [63,64,65,66,67,68,69,70,71,72,73,74,75,76,77,78,79,80,81,82,83,84,85,86,87]. (Created in BioRender. Luca, S. (2025) https://BioRender.com/i42i063, accessed on 9 August 2025).

Since the introduction of CVOTs, new classes of antidiabetic drugs were developed for the treatment of T2DM (Figure 1, Table 1), including the dipeptidyl-peptidase-4 inhibitors (DPP-4i), glucagon-like peptide-1 receptor agonists (GLP-1 RAs), and sodium-glucose cotransporter-2 inhibitors (SGLT2i) [61]. Over the past decade, several CVOTs published showed not only CV safety but also superiority through supplementary CV protection with novel glucose-lowering drugs, especially SGLT2i and GLP-1 RAs [62].

### 6.1. SGLT2i

Regarding the SGLT2i, several major CVOTs support their use in daily practice, with EMPA-REG OUTCOME (2015) being the first trial that investigated the effects of empagliflozin on CV morbidity and mortality in 7020 patients with T2D and established CVDs. The primary endpoint consisted of a 3-point MACE (CV death, non-fatal MI, and stroke) while the secondary endpoint included a composite of hospitalization for unstable angina, HF, and all-cause mortality. The results showed that, in a cohort of T2D patients with established CVDs, treatment with empagliflozin was associated with a significant reduction in the primary composite endpoint (HR = 0.86; *p* = 0.04 for superiority), driven mainly by a reduction in CV deaths (38% relative risk reduction), with no significant differences in MI or stroke rates. In addition to the proven benefits on CV mortality, the drug provided a significantly lower risk in all-cause mortality (32% relative risk reduction) and hospitalization for heart failure (HHF) rates (35% relative risk reduction) compared to placebo [63].

The CANVAS Program (2017) was composed of two sister trials: CANVAS, focused on CV safety, and CANVAS-R, which examined albuminuria progression alongside CV outcomes. The results of the primary analysis were positive, showing a reduction in CV death, non-fatal MI, or stroke (HR = 0.86; *p* < 0.001 for noninferiority; *p* = 0.02 for superiority). However, the trial did not demonstrate superiority for the first secondary outcome, all-cause mortality (*p* = 0.24). Regarding adverse effects, there was an increased risk of limb amputations and bone fractures in the canagliflozin group. Since the secondary endpoints of the trial were not met, renal outcomes in the CANVAS Program were considered exploratory, yet still promising, with a reduction in albuminuria indicating a possible nephroprotective effect of the drug [64].

DECLARE-TIMI 58 (2018) was the first trial to evaluate the CV safety of dapagliflozin and included over 17,000 high-risk patients with T2D (6974 with established CVDs and 10,186 without history of CVDs, but with multiple CV risk factors) that were randomized to receive either 10 mg of dapagliflozin daily or placebo. The trial had a primary safety endpoint consisting of a composite of MACEs and two primary efficacy endpoints that included MACEs and a combination of CV death and HHF incidence. While dapagliflozin proved superior in reducing CV death and HHF rates (HR = 0.83; *p* = 0.005), it was noninferior to placebo in reducing MACEs, not reaching the statistical threshold for superiority (*p* = 0.17) [65].

Ertugliflozin’s safety was explored in the VERTIS CV trial (2020), which included patients with T2DM and established atherosclerotic CV disease (ASCVD). It was tested for noninferiority in the primary endpoint comprising a composite outcome of MACEs and in secondary endpoints like CV death, a composite outcome of CV death and HHF, and a renal composite endpoint (death from renal causes, renal replacement therapy, or doubling of serum creatinine levels). Ertugliflozin proved to be noninferior versus placebo in reducing MACEs in patients with T2D and ASCVD (HR = 0.97; *p* < 0.001 for noninferiority). However, the key secondary composite endpoint, consisting of CV death and HHF, and CV death alone did not differ significantly between treatment groups. Although HHF rates were not statistically tested in the trial, the effects of ertugliflozin were consistent with those observed in previous trials with SGLT2 inhibitors [66].

### 6.2. GLP1-RAs

ELIXA (2015) was the first trial to assess the CV safety of GLP1-RAs. By including patients with T2DM that experienced an acute coronary syndrome (ACS) in the previous 180 days, the trial was designed to evaluate the effect of lixisenatide on the primary composite endpoint of CV death, MI, stroke, or hospitalization for unstable angina. After a follow-up period of 25 months, lixisenatide proved noninferior to placebo regarding the primary composite outcome (HR = 1.02; *p* < 0.001 for noninferiority; *p* = 0.81 for superiority), with comparable safety profiles between the two groups [67].

In the LEADER trial (2015), liraglutide was compared to placebo in a cohort of high-risk patients with T2D with either established CVDs or CV risk factors. The trial’s primary endpoint assessed liraglutide’s superiority versus placebo regarding the incidence of MACEs. Key findings showed a statistically significant reduction in both all-cause mortality and CV death and a numerical reduction in stroke and MI rates, along with a lower rate of MACEs (HR = 0.87; *p* < 0.001 for noninferiority; *p* = 0.01 for superiority). Additionally, treatment with liraglutide indicated potential renal benefits by reducing the incidence of nephropathy events [68].

The first CVOT conducted with semaglutide, SUSTAIN-6 (2016), included mainly patients with established CVDs (83%). Treatment with once weekly injectable semaglutide 1 mg generated a superior reduction in MACE incidence (HR = 0.74; *p* = 0.02 for superiority), mainly driven by a significant decline in stroke rates, as well as a lower incidence and progression of diabetic nephropathy, but with higher rates of retinopathy complications compared to the placebo group [69].

The EXCSEL trial (2017) was designed to demonstrate the CV effects of subcutaneous exenatide in individuals with T2D, both with (73.1%) and without (26.9%) a history of CVDs. Along with the primary composite outcome, a coprimary hypothesis was tested that the drug would demonstrate noninferiority to placebo in terms of safety and superiority to placebo in terms of efficacy. The analysis showed that once-weekly administration of subcutaneous exenatide was noninferior in terms of safety (*p* < 0.001) but failed to demonstrate superiority in terms of efficacy compared to placebo (HR = 0.81; *p* = 0.06), meaning that treatment with exenatide did not significantly reduce the incidence of MACEs [70].

HARMONY OUTCOMES (2018) evaluated albiglutide’s noninferiority to placebo in patients with T2D and CVDs (coronary, cerebrovascular, or peripheral artery disease). The composite primary outcome occurred in 7% of the patients in the treatment group, compared to 9% in the placebo group (HR = 0.78; *p* < 0.0001 for noninferiority; *p* = 0.0006 for superiority), indicating the superiority of injectable albiglutide compared to placebo in reducing MACEs. Regarding the secondary outcomes, only the reduction in MI rates reached statistical significance [71].

In the REWIND trial (2019), a cohort of patients with T2D and established CVDs (31.5%) or additional CV risk factors were randomly assigned to receive subcutaneous dulaglutide or placebo. Along with the primary endpoint, several secondary endpoints were also tested—a composite clinical microvascular outcome (retinopathy and nephropathy), each individual component of the primary outcome, hospitalization for unstable angina, HHF, and mortality rates. In terms of results, subcutaneous dulaglutide proved superior (HR = 0.88; *p* = 0.026) versus placebo in reducing MACEs, primarily by reducing the incidence of nonfatal stroke, in individuals with T2D and high CV risk [72].

After the success of injectable semaglutide, the PIONEER-6 trial (2019) was the first trial that evaluated the CV safety of oral semaglutide. Designed as a noninferiority trial, it included a cohort of 3183 individuals aged above 50 years with established CVDs or chronic kidney disease (CKD) (84.7%) or aged above 60 years with CV risk factors. After a median follow-up period of 15.9 months, results demonstrated that oral semaglutide was noninferior to placebo in reducing the incidence of MACEs (HR 0.79; *p* < 0.001 for noninferiority) [73].

FREEDOM-CVO (2021) investigated the CV safety of ITCA 650, a device that delivers a continuous subcutaneous infusion of exenatide, for the treatment of T2D. The trial population included both patients with CVDs (76%) and CV risk factors only (24%), followed for a short period of 16 months. Primary endpoint was MACEs plus hospitalization for unstable angina. Results showed that ITCA 650 was noninferior (HR = 1.21; *p* = 0.004) compared to placebo in terms of CV safety [74,75].

The latest trial with GLP-1 RAs, SOUL (2025), was designed as a superiority trial and investigated the efficacy of oral semaglutide in a cohort of patients with T2D and established ASCVD (56.6%), CKD (13.1%), or both (27%). Over a longer follow-up period (median 49.5 months), the drug demonstrated superiority versus placebo in reducing MACEs (HR = 0.86; *p* = 0.006), with similar rates of severe adverse events between the two groups, establishing the safety and efficacy of oral semaglutide in this high-risk population [76].

In populations at elevated CV risk, such as individuals with T2D, it is mandatory to adopt strategies that extend beyond glycemic control, the main goal being not only safety but additional CV protection. According to the Framingham Heart Study, patients with T2D are at twice to five times the risk of developing HF, a condition associated with particularly poor outcomes in this cohort [77]. It is now well-established that achieving optimal glycemic control alone is insufficient to reduce the risks of HF-related hospitalizations and mortality [78]. Therefore, dedicated studies have shifted the focus towards strategies that reduce the risk of HF and provide additional renal protection in this high-risk population [79].

### 6.3. DPP4i

Regarding the validation of cardiovascular safety for the DPP-4i, several CVOTs have been conducted following the rosiglitazone incident. The SAVOR-TIMI 53 trial (2013) evaluated saxagliptin in a cohort of patients with T2D and established ASCVD (78.4%) or at high CV risk, testing a composite of MACEs as the primary endpoint, with results showing a neutral effect on MACEs, but with an increase in HHF rates [80]. Given these results, current recommendations still support the (cautious) use of saxagliptin in patients at high risk of developing HF [81]. The EXAMINE trial (2013) evaluated the safety of alogliptin in patients with T2D and recent acute coronary syndrome and yielded similar results in terms of CV safety [82]. In the TECOS (sitagliptin-2015) [83] and CARMELINA (linagliptin-2018) [84] outcome studies, noninferiority of the DPP-4i was confirmed regarding a composite of MACEs (CV death, nonfatal MI, or nonfatal stroke, along with hospitalization for unstable angina in the TECOS trial), but without an increase in HHF. Until this point, no convincing evidence of CV benefits had been seen with the DPP4i class [85].

### 6.4. Insulin

Two CVOTs were conducted with insulin. The ORIGIN trial (2012) included participants aged 50 years or older with either impaired fasting blood glucose levels or T2D who were randomly assigned to receive either insulin glargine or placebo to achieve a target fasting glucose below 95 mg/dL. The co-primary outcomes included MACEs (nonfatal MI, nonfatal stroke, or CV death), along with MACEs combined with revascularization or HHF. Insulin glargine had a neutral effect in terms of CV outcomes, with no significant difference found in the incidence of both coprimary outcomes between treatment groups (HR 1.02; *p* = 0.63 for the first primary outcome vs. HR 1.04; *p* = 0.70 for the second primary outcome). In terms of mortality or microvascular events, results were also similar [86]. The DEVOTE trial (2017) investigated the noninferiority of insulin degludec compared to glargine in terms of CV safety. The cohort consisted of 85.2% patients with established CVDs, CKD, or both. As for the primary composite outcome, the first occurrence of MACEs, results found that insulin degludec was noninferior to insulin glargine with respect to the incidence of MACE (HR 0.91; *p* < 0.001 for noninferiority), with a lower rate of severe hypoglycemia [87].

## 7. The Future Is Bright

The success of the novel glucose-lowering medications on CV outcomes laid the foundation of future perspectives for their use, with trials demonstrating the superiority of SGLT2i in reducing HHF rates and currently emerging as both antidiabetic and cardiovascular drugs, proving safety and efficacy across all spectra of the ejection fraction, regardless of the presence of diabetes [88]. Consistent with findings from CVOTs, dedicated HF trials established the role of dapagliflozin and empagliflozin as a valuable treatment option in patients with HF with reduced ejection fraction (HFrEF), with data from the DAPA HF and EMPEROR-Reduced trials showing a reduction in the composite outcome of hospitalization rates or urgent hospital visit due to HF and CV death in patients already receiving optimal medical therapy [89,90]. Findings from DELIVER and EMPEROR-Preserved extended the use of SGLT2i to HF patients with preserved (HFpEF) and moderately reduced ejection fraction (HFmrEF), thus becoming one of the few treatment options that proved to be efficient in these HF subtypes [91,92].

Driven by observations from previous outcome trials, empagliflozin, dapagliflozin, and canagliflozin were investigated as an emerging therapeutic option in the diverse population of patients with CKD. In CREDENCE, the study group included only patients with T2D, while DAPA-CKD and EMPA-KIDNEY targeted a more heterogenous population, enrolling patients both with and without diabetes. All trials led to similar results in terms of slowing kidney disease progression, highlighting the role of SGLT2i as a safe and efficient treatment option in CKD patients, regardless of the presence or absence of T2D [93].

GLP1-RAs exert anti-atherosclerotic effects by improving both traditional risk factors like weight, lipid profile, and blood pressure, along with direct vascular effects such as improving endothelial function and reducing oxidative stress and inflammation. They have become a veritable anti-atherosclerosis drug class, with research highlighting their potential role in both reducing stroke and MI rates, along with plaque stabilization capacity [94], making them a valuable tool in the therapeutic arsenal of cardiologists.

Although these benefits were already established in patients with T2D, semaglutide 2.4 mg was investigated in the SELECT trial, a CVOT dedicated to patients with established ASCVD but without diabetes, yielding similar results in terms of MACEs [95]. These results have expanded semaglutide’s use to nondiabetic patients with chronic coronary syndrome and a BMI of 27 kg/m^2^ or greater, as recommended by the European Society of Cardiology (ESC) 2024 Guidelines [96].

Current evidence also sustains the use of semaglutide in obesity-related HFpEF, in patients both with and without T2D, with results showing an improvement in symptoms and exercise tolerance, along with significant weight reduction when compared to placebo [97].

In terms of renal benefits in patients with T2D, the aim of the FLOW trial was to determine the effect of semaglutide 1 mg on the progression of CKD. Results demonstrated a significant decrease in kidney outcomes and CV death, along with a slower decline in renal function, lower MACE incidence, and lower mortality rates in patients treated with semaglutide, establishing its role as a potential treatment option in diabetic CKD [98].

## 8. CV Risk Stratification Models: A Timeline

Even if T2D is a well-known risk factor for CVDs, a potential problem in identifying individuals at an imminent risk of developing major CV events is the uneven distribution of CV risk among these patients [99], reflected by the significant variations in disease duration, glycemic and lipid control, blood pressure values, and the presence of comorbidities [100]. While all individuals with T2D are at an increased risk of CVDs compared to the general population, this risk is disproportionately higher in some subgroups [101], indicating the need for more tailored CV risk prediction models that can more accurately stratify patients based on their individual risk, optimize treatment strategies, and facilitate timely interventions, ultimately leading to better outcomes [102].

The Framingham Heart Study (FHS), the first large-scale study that shaped the understanding of the association between diabetes and CVDs [13], highlighted the importance of prevention in high-risk individuals rather than just treating those with established disease by identifying key CV risk factors to better target interventions for prevention and reduce future CV events in patients with T2D [12]. Developed based on data obtained from the FHS, the Framingham Risk Score (FRS) was one of the first tools used to estimate CV risk in the general population and, even though the initial version was not diabetes-specific, subsequent versions incorporated diabetes as a risk factor [103].

The UKPDS Risk Engine was the first diabetes-specific CV risk calculator and included variables such as HbA1c levels, systolic BP values, and cholesterol levels along with other conventional CV risk factors [104].

However, the Framingham and SCORE models are not necessarily reliable in evaluating fatal CVD and coronary heart disease (CHD) risk in T2D patients and therefore are not recommended for use in this specific population [105].

Derived from the ADVANCE trial, the ADVANCE Risk Model can predict the 4–6-year risk of major CV events in T2D patients, and by including diabetes-specific variables like albuminuria and disease duration, aiming at improving risk prediction in this population. This model demonstrated good performance when tested on the ADVANCE population and significantly outperformed the FRS when tested on a multinational cohort with T2DM [99], whereas Framingham and UKPDS risk models could not accurately predict CV risk, highlighting the need for more contemporary and accurate risk assessment tools [106].

Derived from the ACCORD study population, the RECORDe model was developed as an updated risk engine for CV risk stratification in T2D [107] and was compared with the UKPDS risk Engine and ACC/AHA Pooled Cohort Equations. Interestingly, both models overestimated CV risk in the studied population, while the RECORDe equations were more accurate in predicting the risk of micro- and macrovascular complications [108].

The DIAL model was developed to predict 10-year CVD risk along with long-time outcome predictions in individuals with T2D using routine patient data, enabling a more personalized patient care [109]. When applied to a Chinese cohort, the DIAL model overestimated 5-year CV risk but had a better accuracy in predicting 10-year CV risk [110]. An updated, geographically recalibrated DIAL2 model has proven effective in predicting lifetime CVD risk in individuals with T2D with no prior CVDs, particularly in low- and moderate-risk European regions [111].

Different CVD prediction models have been designed for patients with T2D, but none proved to be accurate enough in identifying individuals who experienced a CV event during a 10-year follow-up period, showing a modest discriminative ability [112]. An extensive study revealed that no single prediction model consistently outperformed all others across all CVD endpoints, indicating that a thorough sub-analysis of risk scores is necessary to understand the impact of race, as it led to unexpected outcomes for MI in the analysis [113].

Currently, each set of guidelines recommends a different CVD prediction model, despite limited evidence of external validation across these algorithms [114]. ESC guidelines recommend using diabetes-specific risk models like UKPDS risk engine or the ADVANCE model with caution, as these models are based on older cohort data and may not accurately reflect current conditions [115]. The American Diabetes Association (ADA) suggests that the ASCVD Risk Estimator Plus is a useful tool for assessing CV risk and improving treatment strategies, but by including diabetes as a risk factor, it overlooks other important variables, such as disease duration and complications. The ADA recommends a comprehensive management approach that incorporates established strategies to effectively reduce CV risk [116].

CV risk assessment in recent years has taken a turn to a more practical approach in the 2019 ESC Guidelines on Diabetes, Prediabetes and Cardiovascular Disease, by classifying patients with T2D into three CV risk categories: moderate, high, and very high. Patients under 50 years of age with T2D, with a disease duration of less than 10 years, and no additional risk factors are classified as being at moderate risk. Those with a disease duration of over 10 years and any additional risk factors are considered at high risk. The very high risk category includes patients with T2D who have established CVDs, target organ damage (TOD) (such as proteinuria, eGFR < 30 mL/min/1.73 m^2^, left ventricular hypertrophy, or retinopathy), or over three major risk factors (age, hypertension, dyslipidemia, smoking, and obesity) [117]. An update on CV risk stratification emerged in the 2021 ESC Guidelines, with a change in the definition of severe TOD: eGFR < 45 mL/min/1.73 m^2^ irrespective of albuminuria, eGFR between 45 and 59 mL/min/1.73 m^2^ and microalbuminuria (albumin-creatinine ratio 30–300 mg/g), proteinuria (ACR > 300 mg/g) or the presence of microvascular disease in at least three sites (microalbuminuria, neuropathy, and retinopathy). The revision placed more focus on microvascular complications and less on the presence of additional risk factors. In addition to classifying patients into moderate, high, or very high CV risk categories, the guidelines advise estimating residual 10-year CV risk and evaluating lifetime CVD risk and the potential benefits of risk factor treatment in the high- and very high-risk groups, with the use of the ADVANCE risk score or the DIAL model [115].

The ESC Guidelines on Diabetes and CVD had a major revision in 2023 with the introduction of SCORE2-Diabetes, a novel CV risk calculator tailored for patients with T2D [118]. SCORE2-Diabetes is an algorithm developed from the original SCORE2 model used in the nondiabetic population that includes additional diabetes-specific variables like age at diagnosis, HbA1c levels, and eGFR. The algorithm was calibrated to account for CV mortality rates across Europe by dividing it into four distinct risk regions: low-, moderate-, high-, and very high-risk [119]. Based on the result, patients can be clustered into four different CV risk categories, namely, low, moderate, high, and very high risk, with those who have clinically established ASCVD or severe TOD falling into the very high risk category. Current European guidelines recommend that, in patients over 40 years of age without ASCVD or severe TOD, the 10-year CV risk should be estimated using the novel SCORE2-Diabetes model [120].

Although a wide variety of CV risk models exist for T2D [114], their performance and accuracy remain debatable and, given the substantial variation in CV risk rates across Europe, models like DIAL and ADVANCE are considered geographically limited, lowering their accuracy in estimating CV risk in more diverse populations. And since these models were developed from a narrow set of data, they do not account for current CVD rates. SCORE2-Diabetes is at this point the recommended model for CV risk stratification in T2D [118]. A possible limitation of this algorithm is the extensive data required for calculation, which can be a potential problem in its use in clinical practice, since some of the variables included in SCORE2-Diabetes are not routinely measured. In a real-world setting, an ideal tool would be easier to use and more practical for daily clinical application. A potential issue is whether the endpoints used are sufficient for T2D patients, since the algorithm does not include HF among them. A second concern is whether the included risk factors are enough, given that albuminuria, a well-established risk factor for chronic kidney disease, is excluded from this algorithm. Similarly, the presence of diabetic retinopathy and neuropathy, both recognized predictors of CV risk in T2D, are not included [121].

A comparative study analyzed CV risk stratification in T2D patients using the 2019, 2021, and 2023 ESC Guidelines. The analysis was conducted in a high-risk European population in terms of CV mortality rates. While results showed no significant differences between the 2019 and 2023 guidelines in predicting CV risk, the 2021 guidelines significantly underestimated the CV risk in the cohort, leading to the hypothesis that this classification may be more appropriate for lower-risk European regions. Both the 2019 and 2023 ESC methods accurately estimated CV risk in high-risk populations [118].

## 9. Perspective on Clinical Practice

Over the past decade, T2D management has shifted well beyond the initial glucose-centered approach toward a strategy focused on reducing overall CV risk. After the introduction of CVOTs, major scientific guidelines from the ADA and ESC now recommend prioritizing therapies with proven CV benefits, particularly SGLT2i and GLP-1 RAs, regardless of baseline HbA1c levels or concomitant metformin use. If HbA1c targets are not achieved, combination therapy with both drug classes may be considered for additional glycemic control. Metformin remains an established therapy for T2D due to its efficacy in controlling glycemia and its demonstrated CV safety, and both the ESC and ADA guidelines acknowledge its pivotal role as a historical diabetes medication. However, the European and American guidelines are broadly aligned in their management and treatment strategies in T2D, with emphasis on CV and renal comorbidities, as well as a patient-centered approach [120,122].

In parallel, weight management strategies are evolving rapidly with the development of drug classes like GLP-1 RAs and dual GIP/GLP-1 agonists, highlighting the importance of mitigating obesity as a major driver of T2D and CVDs. Bariatric surgery, however, continues to provide durable benefits and remains a valuable option for patients with a BMI ≥ 35 kg/m^2^, after the failure of lifestyle interventions and pharmacotherapy [120].

Despite these advancements in pharmacotherapy, lifestyle interventions remain fundamental in T2D management, with both the European and American guidelines strongly advising for dietary changes and physical activity as first-line interventions [120,122]. Structured patient education and provider counseling improve patient engagement in self-care, possibly translating to better long-term outcomes [123].

Growing evidence also supports the role of specialized nurses as part of an integrated multidisciplinary care team, helping in improving glycemic control and quality of life in diabetes care [124,125].

Along with lifestyle and pharmacological interventions, nutraceuticals can play a potential complementary role in the management of T2D. Coenzyme Q10 supplementation can modestly lower glycemic and lipid parameters, therefore aiding in the mitigation of cardiometabolic risk factors [126]. Research is ongoing in the nutraceutical field, with emerging evidence suggesting that supplements, when combined with dietary interventions, may help improve metabolic control; however, current evidence supports their use only as an adjunctive intervention in T2D management [127].

## 10. Limitations

This article is a narrative review and is, therefore, not intended to provide a systematic synthesis of all the available literature. Articles were selected for their relevance to the historical timeline of the association between T2D and CVDs. We also acknowledge the potential for selection bias and the inclusion of older research articles to provide historical context.

## 11. Conclusions

CVD is a well-known and feared complication of T2D, significantly increasing morbidity and mortality rates, and the individual assessment of CV risk plays a crucial role in managing these patients. History remains a valuable teacher, showing that glycemic control, although important, is not the cornerstone of DM management anymore. Early landmark trials revealed that aggressive glucose-lowering strategies alone were insufficient to reduce macrovascular complications, particularly CVDs. The controversy surrounding rosiglitazone raised safety concerns and prompted a regulatory shift mandating the introduction of CVOTs. Since then, the treatment paradigm underwent a major shift with the introduction of novel glucose-lowering medications such as iSGLT2 and GLP-1 RAs, taking diabetes treatment from the initial glucocentric approach towards not only CV and renal safety, but ultimately protection, fundamentally changing guideline recommendations. By tracing these milestones, this review highlights the evolution towards a comprehensive and multidisciplinary approach to mitigating CV risk in T2D management. Future research will continue to drive the focus towards optimizing CV outcomes in patients with T2D, offering potential for more targeted and effective interventions.

## Figures and Tables

**Table 1:** CVOTs of novel glucose-lowering drugs [63,64,65,66,67,68,69,70,71,72,73,74,75,76,77,78,79,80,81,82,83,84,85,86,87].

Drug Class	Trial (Year) Drug Investigated	Population	Primary Endpoint	Key Results	Notable Adverse Effects
***SGLT2*** ***inhibitors***	EMPA-REG OUTCOME (2015) *empagliflozin*	7020 T2D + CVD	3-point MACE	↓ MACE (HR 0.86), ↓ CV death, ↓ all-cause mortality, ↓ HHF	Genital infections
	CANVAS Program (2017) *canagliflozin*	10,142 T2D + high CV risk	3-point MACE	↓ MACE (HR 0.86); exploratory renal benefit	↑ Amputations, fractures
	DECLARE-TIMI 58 (2019) *dapagliflozin*	17,160 T2D (40% CVD)	3-point MACE	neutral MACE (noninferior)	Genital infections
	VERTIS-CV (2020) *ertugliflozin*	8246 T2D + ASCVD	3-point MACE	Noninferior	Genital infections
***GLP-1*** ***RAs***	ELIXA (2015) *lixisenatide*	6068 T2D + recent ACS	3-point MACE + hospitalization for unstable angina	Noninferior	GI events
	LEADER (2015) *liraglutide*	9340 T2D + high CV risk	3-point MACE	↓ CV death, ↓ MACE (HR 0.87)	Gl events, gallbladder discase
	SUSTAIN-6 (2016) *Semaglutide (subcutaneous)*	3297 T2D + CVD/CKD/CV risk factors	3-point MACE	↓ MACE (HR 0.74): ↓ stroke	Retinopathy risk, GI events
	EXSCEL (2017) *exenatide*	14,752 T2D (73% CVD)	3-point MACE	Noninferior	GI events
	HARMONY OUTCOMES (2018) *albiglutide*	9463 T2D + CVD	3-point MACE	↓ MACE (HR 0.78)	GI events
	REWIND (2019) *dulaglutide*	9901 T2D (31.5% CVD)	3-point MACE	↓ MACE (HR 0.88): ↓ stroke	GI events
	PIONEER-6 (2019) *Semaglutide (oral)*	3183 T2D (85% CVD/CKD)	3-point MACE	Noninferior	GI events
	FREEDOM-CVO (2021) *ITCA650*	>4000 T2D (76% CVD)	Expanded MACE	Noninferior	Device issues
	SOUL (2025) *Semaglutide (oral)*	>9000 T2D + ASCVD/CKD	3-point MACE	↓ MACE (HR 0.86)	GI events
***DPP-4*** ***inhibitors***	SAVOR-TIMI 53 (2013) *saxagliptin*	16,492 T2D + ASCVD/high risk	3-point MACE	Neutral; ↑ HHF	↑ HHF risk
	EXAMINE (2013) *alogliptin*	5380 T2D + ACS	3-point MACE	Neutral	Similar to placebo
	TECOS (2015) *sitagliptin*	14,671 T2D + CVD	4-point MACE	Neutral	Similar to placebo
	CARMELINA (2018) *linagliptin*	6979 T2D + CVD/CKD	3-point MACE	Neutral	Similar to placebo
***Insulin***	ORIGIN (2012) *glargine*	12,537 T2D/prediabetes + risk factors	3-point MACE; MACE + revascularization/HHF	Neutral	Hypoglycemia
	DEVOTE (2017) *degludec*	7637 T2D (85% CVD/CKD)	3-point MACE	Noninferior to glargine	Hypoglycemia

## Abbreviations

The following abbreviations are used in this manuscript:

ACS	Acute coronary syndrome
ADA	American Diabetes Association
ASCVD	Atherosclerotic cardiovascular disease
CHD	Coronary heart disease
CI	Confidence interval
CKD	Chronic kidney disease
CVD	Cardiovascular disease
CVOT	Cardiovascular outcome trial
DPP4i	Dipeptidyl peptidase-4 inhibitor
ESC	European Society of Cardiology
FHS	Framingham Heart Study
FRS	Framingham Risk Score
GLP-1 RAs	Glucagon-like peptide-1 receptor agonists
HF	Heart failure
HFmrEF	Heart failure with moderately reduced ejection fraction
HFpEF	Heart failure with preserved ejection fraction
HFrEF	Heart failure with reduced ejection fraction
HHF	Heart failure hospitalization
MACE	Major adverse cardiovascular event
MeS	Metabolic syndrome
MI	Myocardial infarction
RCT	Randomized controlled trial
RR	Risk ratio
SGLT2i	Sodium-glucose transport protein 2 inhibitors
T2D	Type 2 diabetes
TOD	Target organ damage
